# Comparative pathology of pigs infected with Korean H1N1, H1N2, or H3N2 swine influenza A viruses

**DOI:** 10.1186/1743-422X-11-170

**Published:** 2014-09-24

**Authors:** Kwang-Soo Lyoo, Jeong-Ki Kim, Kwonil Jung, Bo-Kyu Kang, Daesub Song

**Affiliations:** Korea Zoonosis Research Institute, Chonbuk National University, Iksan, Jeonbuk, 561-756 Republic of Korea; College of Pharmacy, Korea University Sejong Campus, 2511 Sejong-Ro, Sejong City, 339-700 Republic of Korea; Ohio Agricultural Research and Development Center, Department of Veterinary Preventive Medicine, The Ohio State University, Wooster, OH 43210 USA; Research Unit, Green Cross Veterinary Products, Yong-in, 449-903 Republic of Korea; Viral Infectious Disease Research Center, Korea Research Institute of Bioscience and Biotechnology, Daejeon, 305-806 Republic of Korea

**Keywords:** Comparative pathogenesis, Swine influenza virus, Pneumonia, Pathology, Subtypes

## Abstract

**Background:**

The predominant subtypes of swine influenza A virus (SIV) in Korea swine population are H1N1, H1N2, and H3N2. The viruses are genetically close to the classical U.S. H1N1 and triple-reassortant H1N2 and H3N2 viruses, respectively. Comparative pathogenesis caused by Korean H1N1, H1N2, and H3N2 SIV was evaluated in this study.

**Findings:**

The H3N2 infected pigs had severe scores of gross and histopathological lesions at post-inoculation days (PID) 2, and this then progressively decreased. Both the H1N1 and H1N2 infected pigs lacked gross lesions at PID 2, but they showed moderate to severe pneumonia on PID 4, 7 and 14. The pigs infected with H1N1 had significant scores of gross and histopathological lesions when compared with the other pigs infected with H1N2, H3N2, and mock at PID 14. Mean SIV antigen-positive scores were rarely detected for pigs infected with H1N2 and H3N2 from PID 7, whereas a significantly increased amount of viral antigens were found in the bronchioles and alveolar epithelium of the H1N1infected pigs at PID 14.

**Conclusions:**

We demonstrated that Korean SIV subtypes had different pulmonary pathologic patterns. The Korean H3N2 rapidly induced acute lung lesions such as broncho-interstitial pneumonia, while the Korean H1N1 showed longer course of infection as compared to other strains.

## Background

Swine influenza virus (SIV) is an important global pathogen in the swine industry. Pigs affected by SIV usually show an abrupt outbreak of respiratory symptoms characterized by abdominal breathing and coughing, fever, depression, and increased mortality due to secondary bacterial infections [1]. Morbidity may be exhibited at 100% in SIV-infected swine herd, although mortality in the pigs is low [2]. In histopathological terms, the influenza virus infection in pigs is characterized by necrosis of bronchial and bronchiolar epithelial cells during the initial phases of infection. This is accompanied by the accumulation of necrotic debris and neutrophils in the bronchial and bronchiolar lumina [3].

The three main subtypes of SIV are H1N1, H1N2, and H3N2, all of which express multiple antigenic determinants and are presently circulating in the swine population of North America, Europe, and Asia [4]. Since the first SIV was recognized in 1930, the classical swine lineage H1N1 has been relatively stable at the genetic and antigenic levels in the U.S. swine population [5]. In 1998, the swine influenza viruses in U.S. pigs underwent considerable genetic changes that led to the emergence of the so-called triple-reassortant H3N2 [4, 5]. The triple-reassortant H3N2 consists of gene segments from the classical swine virus (M, NP and NS), H3N2 human seasonal virus (HA, NA and PB1), and avian influenza virus (PA and PB2) [5]. Subsequently, in 1999, a second-generation reassortant H1N2 containing genes derived from the triple-reassortant H3N2 and classical swine influenza H1N1 was isolated in U.S. pigs [2].

In Korea, a number of researchers have demonstrated that the predominant SIVs in the swine population are H1N1, H1N2, and H3N2, despite pandemic H1N1/2009 being recently identified in Korean pigs [6–9]. According to both serologic and genetic analysis, the three SIV subtypes from Korea are very similar to the classical swine lineage or the triple-reassortant viruses from the U.S. [8, 10]. Although the pathological characteristics of each individual SIV subtype have been investigated, no studies have been conducted to demonstrate the pathogenesis of all three subtypes together. We therefore compared the pathological features of Korean H1N1, H1N2, and H3N2 SIV infection in pigs.

## Methods

### Viruses

An H1N1 (A/swine/Korea/GC0503/05) is genetically close to the representative classical swine influenza lineage from the U.S. [8]. While H1N2 (A/Swine/Korea/6822/06) and H3N2 (A/Swine/Korea/GC0407/05) are genetically similar to the triple-reassortant viruses from the U.S.[8]. All three viruses isolated from lung samples of six to eight-week-old pigs with acute respiratory problems and were propagated in Madin-Derby canine kidney (MDCK) cells grown in TPCK-trypsin (2 μg/ml)-treated Eagle’s minimal essential medium (MEM, GIBCO/BRL). Following 10-fold serial dilutions of the virus stocks, each virus was quantified by analysing its growth in MDCK cells [11].

### Study design

Sixty (3-weeks-old) Large White-Duroc crossbred pigs were obtained from a conventional pigs farm, with each pig being serologically negative for porcine reproductive and respiratory syndrome virus (PRRSV), porcine respiratory coronavirus (PRCV), porcine circovirus type 2 (PCV2), and swine influenza H1 and H3 antigens. Pigs were randomly assigned to four groups, and physical examinations were performed to assess the health of the animals prior to virus inoculation. The pigs were then sedated with an intramuscular injection of Telazol® (6 mg/kg, Fort Dodge, Iowa) and Xylazine (1 mg/kg, Akom Inc. IL). Each of the fifteen pigs were then inoculated intratracheally with 3 ml of tissue culture fluid containing 1.0 × 10^6^ tissue culture infectious dose (TCID)_50_/ml of H1N1, H1N2, and H3N2. The remaining 15 pigs were inoculated with just cell culture medium as a mock-infected control. Between post-inoculation days (PIDs) 0 to 21, the pigs were assessed for clinical symptoms, and their respiratory rate and rectal temperature were measured daily. All experimental procedures were approved by an independent Animal Care and Use Committee and followed the guidelines of the Korea Research Institute of Bioscience and Biotechnology.

### Virus detection and pathological examination

Three pigs from each group were euthanized at PIDs 2, 4, 7, 14, and 21; lung tissues were collected for examination following the virus replication in the lung and gross and histological change. To provide the virus replication in pig lungs, viral RNA was extracted from lung sample of all pigs, and RT-PCR was conducted as previously described protocol [8]. To determine macroscopic examination, lung lesions were scored using Madec’s grid according to the method described by Madec and Kobisch [12, 13].Lesions were scored on the area in all six lobes that were occupied by virus-induced lesions as follows (expressed as a percentage of the total area): 0 = no lesions (0%), 1 = mild pneumonia (ranging from 1 to 25%), 2 = moderate pneumonia (ranging from 26 to 50%), 3 = severe pneumonia (ranging from 51 to 75%), and 4 = very severe pneumonia (ranging from 76% to 100%). The sum of the overall score for the each lobe was averaged to reflect the gross lesion of an individual pig. For histopathological examination, tissue sections stained with hematoxylin and eosin (H&E) were blindly evaluated by a pathologist. The lung tissues were scored as previously described with slight modifications [3], as follows: (1) severity of pulmonary epithelial cell necrosis (ranging from 0 to 6); (2) infiltration of inflammatory leukocytes (ranging from 0 to 3); (3) thickening of alveolar septa due to infiltration of inflammatory leukocytes (ranging from 0 to 3); (4) pulmonary haemorrhage and edema (ranging from 0 to 3); and (5) atelectasis due to airway plugging or perivascular inflammation (ranging from 0 to 3). The final score was estimated by adding the score of each criterion.

### Immunohistochemistry

To detect the influenza virus antigens in the lungs, immunohistochemistry (IHC) was performed on all the tissue samples using a previously described protocol [14]. Briefly, tissue slides were incubated with goat polyclonal antibody against the whole anti-influenza A virus (1:100; Chemicon, CA, USA) conjugated with streptavidin-alkaline phosphatase (1:200; Dako, CA). Each section was then counterstained with Mayer’s hematoxylin. Influenza A virus antigen-positive scores were calculated by estimating the number of antigen-positive cells in the lung per microscopic area at 200× magnification. This was based on the following criteria; 0 = no positive cells, 1 = minor (less than 10 positive cells), 2 = moderate (from 11 to 25 positive cells), and 3 = high (more than 26 positive cells).

### Statistical analysis

The SEM (standard error of the mean) was calculated for each value. The data from all four treatment groups (H1N1, H1N2, H3N2, and control) were analyzed by the nonparametric Kruskal-Wallis test using Statistical Analysis Systems software (SAS Institute Inc., Cary, NC). A value of *P* < 0.05 was considered statistically significant.

## Results

All the inoculated pigs showed typical clinical symptoms, namely, sneezing and coughing. They also developed a high fever, and their average rectal temperature exceeded 40.0°C between PID 2 and 4. In contrast, control pigs exhibited no clinical symptoms throughout the experiment. The three groups infected with H1N1, H1N2, and H3N2-infected groups did not differ significantly in their clinical appearance that included sneezing, coughing, rectal temperature, and respiratory rate (data not shown).

The viral RNA of H1N1 was detected in lung samples from the infected pigs at PID 2 (2/3; number of positive pigs/number of pigs tested), PID 4 (3/3), PID 7 (2/3), and PID 14 (1/3), while the viral RNA was detected in the pigs infected with H1N2 or H3N2 at PID 2 (1/3 or 3/3), PID 4 (3/3 or 3/3), and PID 7 (2/3 or 2/3), respectively. However, pigs of the control group remained negative for the viral RNA throughout the study period.

**Figure 1:**
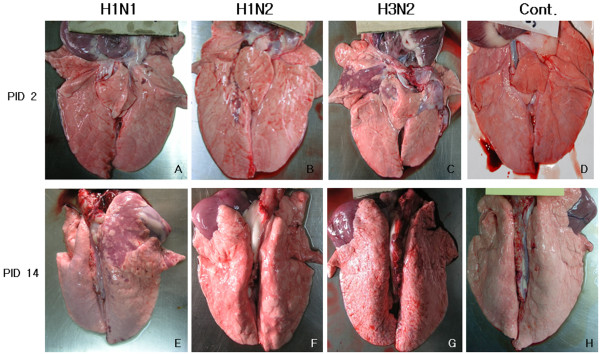
**Gross lung lesion of pigs infected with H1N1, H1N2, or H3N2 and non-infected pigs at post-inoculation days (PID) 2 and 14.** The lesion typical for SIV presents as purple to dark red consolidated area in right cranial lobe of pig infected with H3N2 at PID 2 **(C)**, however the lesion is not shown in the lung of the pig at PID 14 **(G)**. The pig infected with H1N1 shows no lesion at PID 2 **(A)**, but developed purple colored consolidated area in the right cranial lobe at PID 14 **(E)**. The pigs infected with H2N1 shows had a small portion of purple consolidation in right caudal lobe at PID 2 **(B)**, and mild broncho-pneumonia in both caudal lobes at PID 14 **(F)**. Control pigs show no gross lesion at PID 2 **(D)** and at PID 14 **(H)**.

**Figure 2:**
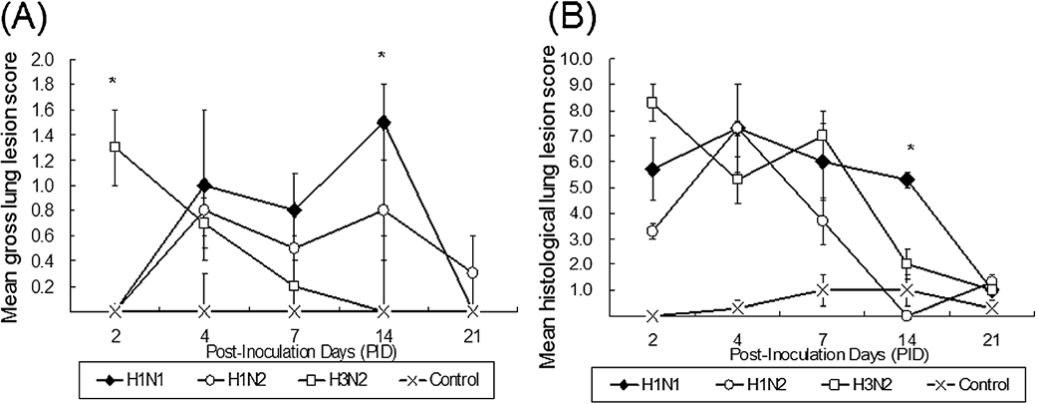
**Mean gross and histological lung lesion scores for the four experimental pig groups at each different PID. (A)** The H3N2-infected pigs had severe pneumonic lesions at PID 2, while H1N1- and H1N2-infected pigs lacked gross lesions; however, at PID 14, the lesion score of H1N1-infected pigs were significantly higher (*P* < 0.05) than those of H1N2- and H3N2-infected pigs. **(B)** The H3N2-infected pigs had higher histological lesion scores than H1N1- and H1N2-infected pigs at PID 2; reversely, at PID 14, the score of H1N1-infected pigs were significantly higher (*P* < 0.05) than those of H1N1 and H3N2 pigs.

The gross lung lesions of infected pigs ranged from multifocal lesions to a coalescing reddish-tan consolidation of the lung, particularly affected the cranial lobes, but these lesions were not detected in the control pigs (Figure 1). The three infected groups differed in their gross lesion scores following post inoculation. The H3N2 infected pigs showed significantly higher lung lesion score than other groups (*P* < 0.05) at PID 2, and this then progressively decreased. Both the H1N1 and H1N2 infected pigs lacked gross lesions at PID 2, but they showed minimal to mild pneumonia lesions on PID 4, 7 and 14. The pigs infected with H1N1 had significant scores (*P* < 0.05) of gross lesions when compared with the other pigs infected with H1N2, H3N2, and control at PID 14 (Figure 2A.).

**Figure 3:**
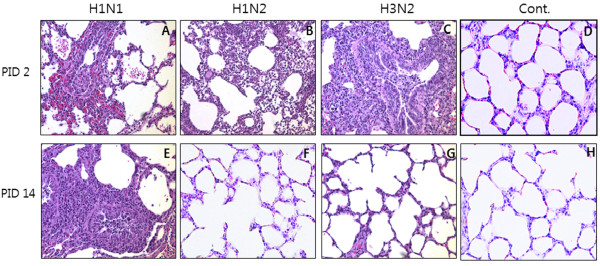
**Histopathological lesion of the lung of the pigs infected with H1N1, H1N2 or H3N2 and non-infected pigs at post-inoculation days (PID) 2 and 14.** The lung section of pig infected with H3N2 shows diffuse necrosis of bronchiolar epithelium and infiltration of inflammatory cells composed of mostly neutrophils and mononuclear cells around bronchioles **(C)**, however most lesions were restored in the pigs at PID 14 **(G)**. The pig infected with H1N1 shows less inflammation around bronchioles, but exhibits hemorrhagic lesions at PID 2 **(A)**, whereas chronic inflammation with lymphocytic infiltration and epithelial proliferation is observed in H1N1-infected pig at PID 14 **(E)**. The pig infected with H1N2 shows mild interstitial pneumonia lesion at PID 2 **(B)**, but the lesion was restored at PID 14 **(F)**. Control pigs show no histopathologic lesion at PID 2 **(D)** and at PID 14 **(H)**.

The histological lesions were limited to the bronchi, bronchioles, and alveoli, but no lesions were observed in the tissues of control pigs. The pigs infected with H1N1, H1N2, and H3N2 all shared the following histopathological features; bronchial/bronchiolar necrosis, thickening of the alveolar septa due to the infiltration of inflammatory leukocytes, and pulmonary hemorrhage and atelectasis (Figure 3). The H3N2 infected pigs had severe histological lesions at PID 2, while, in the other sub-type infected pigs, the lesions were milder at PID 2. However, at PID 14, these lung lesions of H1N1-infected pigs had a significantly higher mean histological score (5.3, *P* < 0.05) than those from the lungs of H1N2 (0) and H3N2 (2.1) infected pigs (Figure 2B).

**Figure 4:**
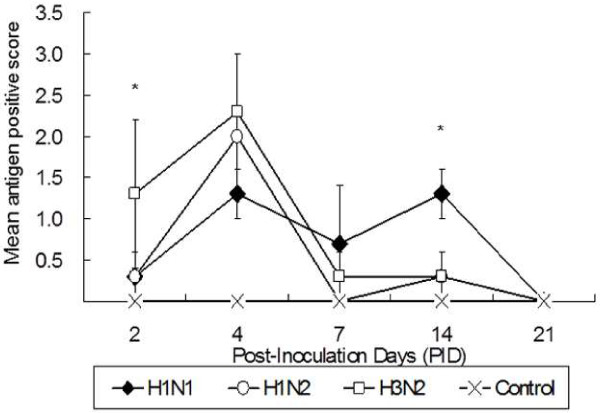
**IHC staining scores at each different PID.** Mean antigen-positive score of the viral antigens of H3N2-infected pigs (*P* < 0.05) was higher than those of the other group’s pigs at PID 2. However, the viral antigen numbers of the H1N1-infected pigs significantly higher (*P* < 0.05) than those of H1N2- and H3N2-infected pigs at PID 14.

**Figure 5:**
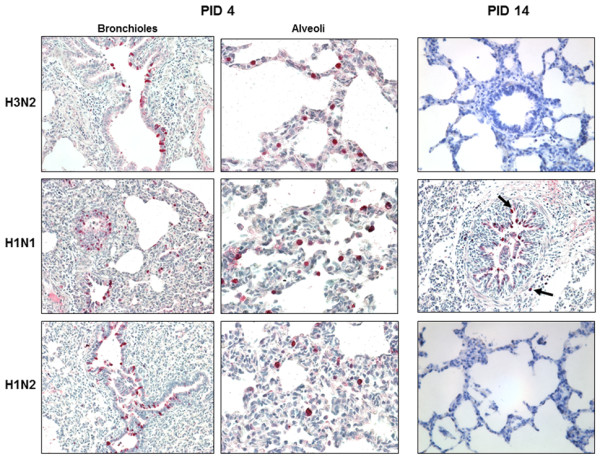
**Localization of influenza A virus antigens in the lungs of pigs with H1N1, H1N2, and H3N2 infection.** Influenza A virus antigens (red stain) were observed in bronchiolar epithelial cells and alveolar macrophage-like mononuclear cells within mildly to moderately thickened alveolar septa at PID 4. At the late stage of infection (PID 14), influenza virus antigens (solid-headed arrow) were observed in H1N1-infected pigs but not for H1N2 and H3N2-infected pigs.

SIV was detected in the lung tissue following IHC staining. Influenza viral antigens were predominantly found in the bronchial and bronchiolar epithelium. In addition, numerous necrotic epithelial cells that underwent phagocytosis by alveolar macrophages were detected in the lumen. In the three infected groups, mean antigen-positive score of the viral antigens of H3N2-infected pigs was higher than those of the other group’s pigs at PID 2. The mean antigen scores of pigs infected with H1N2 and H3N2 were rarely detected from PID 7. However, an increased amount of viral antigens in H1N1infected pigs were found in the bronchioles and alveolar epithelium, and the mean score was significantly higher (*P* < 0.05) than those of H1N2 and H3N2 infected pigs at PID 14 (Figures 4 and 5).

## Discussion

In the present study, we have shown that H3N2 SIV cause massive damage to the bronchial and bronchiolar epithelial cells at the start of infection (PID 2) eliciting gross and histological lesions. The inflammation caused by H3N2 may affect the initial stages of infection. In comparison, H1N1 or H1N2 infected pigs exhibited few (histological) or no (gross) pathological changes at this stage. These results are linked to the mean antigen-positive scores from infected pigs, which demonstrated that the viral antigens were not as widely distributed in the lungs of pigs infected with H1N1 or H1N2 at PID 2 when compared to H3N2 infected pigs. Although the H1N1detection in a pig’s lung of three pigs at PID 14 might be caused by second infection between pigs within the same group, no virus was observed by IHC and RT-PCR in other pigs infected with H1N2 or H3N2.

It is acknowledged that bronchial and bronchiolar epithelial cells and leucocytes found in inflammatory lesions are the main source of pro-inflammatory cytokines [14, 15]. Thus, tissue damage may be responsible for the severe suppurative, necrotizing bronchitis and broncho-interstitial pneumonia observed at the beginning of infection (PID 2) in H3N2-infected pigs.

Subsequently, between PID 4 and 7, inflammation with lymphocytic infiltration and epithelial proliferation were found in the lung tissues from pigs infected with H1N1, H1N2, and H3N2. Influenza viral antigens were also frequently found in the alveolar macrophages within the thickened alveolar septa and the peribronchiolar lymphatic vessels. The gross and histological pulmonary lesions from pigs infected by H1N2 or H3N2 gradually decreased between PID 4 and PID 14, and the mean antigen-positive scores decreased in the lungs from both H1N2 and H3N2 at PID 7. However, the pigs infected with H1N1 SIV showed significantly high levels of the viral antigen distribution, as well as increased gross and histopathological lung scores.

Based on these data, we have demonstrated that H1N1 SIV, isolated from Korea, may replicate in the lung and can induce pneumonic lesions for a relatively longer period than the other two viruses; H1N2 and H3N2. This comparative investigation of SIV isolates from Korea is the first study of their pathology, since the pathogenesis of SIV has only been studied in pigs experimentally infected by each SIV subtype in Korea [3, 16, 17].

Here, our pathological study demonstrated that Korean SIV subtypes have different pulmonary pathologic patterns. The H3N2 rapidly induces acute lung lesions, such as broncho-interstitial pneumonia, while the H1N1 is associated with pulmonary lesions later in infection. This may reflect differences in the speed of initial replication rates in bronchial and bronchiolar epithelial cells. These observations also demonstrate the importance of comparing the pathogenesis of influenza viruses over the entire course of an infection as opposed to an individual time-point.
